# An internal pilot design for prospective cancer screening trials with unknown disease prevalence

**DOI:** 10.1186/s13063-015-0951-3

**Published:** 2015-10-13

**Authors:** John T. Brinton, Brandy M. Ringham, Deborah H. Glueck

**Affiliations:** Denver Health Medical Center, 777 Bannock St., MC 6551, Denver, Colorado 80204 USA; Department of Biostatistics and Informatics, Colorado School of Public Health, University of Colorado Anschutz Medical Campus, 13001 E. 17th Place, Aurora, Colorado 80045 USA

**Keywords:** Cancer screening, Internal pilot area under the curve, Type I error, Power, Receiver operating characteristic analysis

## Abstract

**Background:**

For studies that compare the diagnostic accuracy of two screening tests, the sample size depends on the prevalence of disease in the study population, and on the variance of the outcome. Both parameters may be unknown during the design stage, which makes finding an accurate sample size difficult.

**Methods:**

To solve this problem, we propose adapting an internal pilot design. In this adapted design, researchers will accrue some percentage of the planned sample size, then estimate both the disease prevalence and the variances of the screening tests. The updated estimates of the disease prevalence and variance are used to conduct a more accurate power and sample size calculation.

**Results:**

We demonstrate that in large samples, the adapted internal pilot design produces no Type I inflation. For small samples (*N* less than 50), we introduce a novel adjustment of the critical value to control the Type I error rate. We apply the method to two proposed prospective cancer screening studies: 1) a small oral cancer screening study in individuals with Fanconi anemia and 2) a large oral cancer screening trial.

**Conclusion:**

Conducting an internal pilot study without adjusting the critical value can cause Type I error rate inflation in small samples, but not in large samples. An internal pilot approach usually achieves goal power and, for most studies with sample size greater than 50, requires no Type I error correction. Further, we have provided a flexible and accurate approach to bound Type I error below a goal level for studies with small sample size.

## Background

Lingen et al. [1] proposed a study to compare the diagnostic accuracy of two screening modalities for the detection of oral pre-malignant and malignant lesions. During the planning phase of the trial, Lingen et al. considered a paired design with the full area under the receiver operating characteristic curve (AUC) as the outcome.

In a paired cancer screening trial, each participant is given two screening tests [1–4]. The participants are typically volunteers drawn from a standard screening population. Thus, the trial includes both participants with disease and participants without disease. At entry, the disease status of the participants is unknown. Presumably, the disease status of the participants in the trial mirrors the prevalence in the population.

The sample size for the trial proposed by Lingen et al. depended on the prevalence of disease in the population. The reported prevalence of oral malignant and pre-malignant lesions varied by as much as 16.5 % [5], even in published reports, depending on the population studied. If the prevalence of lesions was 12.1 %, as observed by [5], 2,450 participants would have been required to achieve 95 % power for the trial. However, if the prevalence of lesions was 0.2 % [6], Lingen and his colleagues would have needed to recruit 116,100 participants, a 47-fold increase.

All researchers have an ethical responsibility to choose an accurate sample size. Participants in cancer screening trials may face emotional and physical harm from needless biopsy, false positive diagnoses, and over-diagnosis of non-fatal disease. A study that overestimates the sample size required for a cancer screening trial exposes study participants to needless harm. A study that underestimates the sample size lacks the power to answer the research question, while still exposing study participants to potential harm.

One possible solution to the ethical dilemma is an internal pilot study. In an internal pilot design, investigators use information from the first fraction of study participants accrued to estimate unknown parameters [7–10]. The estimates can then be used to calculate an updated sample size.

Previous work on internal pilot designs for screening studies has assumed that the ratio of cases is known prior to the start of the study and that the ratio is fixed throughout the course of the study. Wu et al. [11] proposed an internal pilot approach for the comparison of the diagnostic accuracy of screening tests, but, like Coffey and Muller [12], assumed that the ratio of cases to non-cases was known before the study, and fixed by design during the study. In addition, the method of Wu et al. [11] does not control for possible Type I error inflation. While Gurka et al. [13] considered the use of internal pilot designs for observational studies, they did not suggest any Type I error correction techniques. In general, in small samples, internal pilot designs can inflate Type I error [14]. There are multiple approaches for controlling Type I error inflation in internal pilots, when the inflation occurs due to variance re-estimation [12, 15–18].

We broaden the definition of the internal pilot design to match the sampling scheme in cancer screening trials. We adapt internal pilot methodology to the cancer screening setting by: 1) allowing the ratio of cases to non-cases to vary randomly throughout the study, 2) re-estimating the sample size with internal pilot sample estimates of both the disease prevalence and the variance of the outcome, and 3) adjusting the critical value to control for possible Type I error rate inflation caused by sample size re-estimation. The critical value correction depends on the unconditional distribution of the test statistic. We show that the approach allows investigators to attain a targeted power level, and control Type I error rate inflation in small samples. We demonstrate, via simulation, that no correction is needed for large samples. The internal pilot approach is applied to two oral cancer screening examples: one small one, where the correction is needed, and one large one, where no correction is needed. We conclude the manuscript with a discussion of the results.

## Methods

### Study design, hypothesis test, and sample size re-estimation

#### A novel internal pilot study design for screening trials

The novel internal pilot design includes the following steps:

1. **Initial planning stage**: Initial estimation of the sample size needed.
2. **Pilot stage**: Collection of paired screening test scores from a fraction of the planned sample size.
3. **Re-estimation**: Sample size re-estimation using pilot-sample based variance and prevalence estimates.
4. **Additional data collection**: Collection of additional data based on the sample size re-estimation.
5. **Analysis**: Hypothesis testing, using an adjusted critical value to prevent Type I error inflation.

We expand the notation of Coffey and Muller [9, 12] and Coffey et al. [19] to accommodate our modifications in the internal pilot study design. Throughout the manuscript lower case letters represent fixed variables and upper case letters represent random variables. Matrices are written in bold text.

**Fig. 1:**
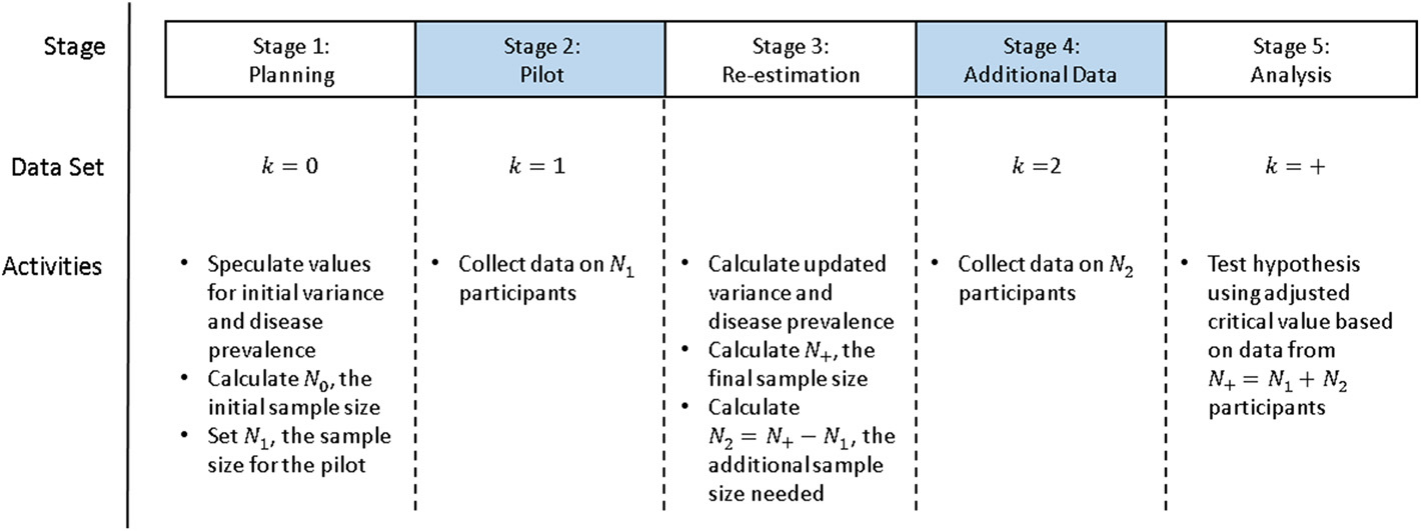
Adapted internal pilot design for a cancer screening study

Data for the internal pilot study can be organized into four sets according to the stage of the study that is of interest (Fig. 1). Let *k* ∈ {0, 1, 2, +} index the stage of interest. Variables indexed by *k* = 0 describe the initial planning stage. Since no data has been collected, planning stage variables take on planned or speculated values. Variables indexed by *k* = 1 and *k* = 2 identify data observed in the pilot stage and the additional data collection stage, respectively. Variables indexed by *k* = + describe the entire sample, which includes data from all participants.

Let the random variable *N*_*dk*_ be the number of study participants in stage *k* with disease status *d* ∈ {*n*, *c*}, with *n* indicating no disease, and *c* disease. For example, *N*_*c*1_ is the number of individuals with disease in the pilot sample. When the subscript *d* is dropped, the random variable *N*_*k*_ denotes the number of people both with and without disease in the *k*th stage of the study. For example, *N*_1_ is the total number of individuals in the pilot sample, and *N*_+_ is the final sample size.

Let *N*_min_ and *N*_max_ be the minimum and maximum sample sizes allowed by the study investigator, and assume that *N*_+_ ∈ [min (*N*_1_, *N*_min_), *N*_max_]. Let *n*_0_ be the initial sample size estimate, and define *λ* = *n*_1_/*n*_0_. Let ${\gamma}_{\pi }=\pi /{\widehat{\pi}}_0$, where *π* ∈ (0, 1) is the true prevalence of disease, and ${\widehat{\pi}}_0\in \left(0,1\right)$ is the initial estimate of prevalence of disease. Let ${\widehat{\pi}}_1={n}_{c1}/{n}_1$ be the estimate of prevalence of disease from the pilot data. With *σ*^2^ the true variance of the difference in the two screening test scores, and ${\widehat{\sigma}}_0^2>0$ the variance estimate used for the initial sample size calculation, define $\gamma ={\sigma}^2/{\widehat{\sigma}}_0^2$. Let ${\mathrm{SSE}}_1={\widehat{\sigma}}_1^2\times \left({n}_1-2\right)$ where ${\widehat{\sigma}}_1^2$ represents the variance of the difference in the two screening test scores estimated after the internal pilot study. Let *P*_*t*_ and *α*_*t*_ be the target power and Type I error level for the study. 

#### A paired comparison of the diagnostic accuracy of two screening tests

Let *y*_*idj*_ be the screening test score for individual *i* ∈ {1, 2, …, *N*_+_}, with disease status *d*, on screening test *j* ∈ {*A*, *B*}. Assume that the two screening test scores [*y*_*idAk*_*y*_*idBk*_]^'^ have a bivariate normal distribution with mean ***μ***_*d*_ = [*μ*_*dA*_*μ*_*dB*_]^'^, *V*(*y*_*idjk*_) = *σ*_*dj*_^2^, and *Cov*(*y*_*idAk*_, *y*_*idBk*_) = *ρ*_*d*_*σ*_*dA*_*σ*_*dB*_. We assume that differences between the screening test scores for both the cases and non-cases are distributed with equal variance, *V*(*y*_*inA*_ − *y*_*inB*_) = *V*(*y*_*icA*_ − *y*_*icB*_) = *σ*^2^. Under the bivariate normal assumption, the AUC for screening test *j* is given by Φ[(*μ*_*cA*_ − *μ*_*nA*_)/*σ*] ([20], p. 83, Result 4.8) where Φ is the cumulative distribution function of the standard normal. The difference between the AUCs is given by Φ[(*μ*_*cA*_ − *μ*_*nA*_)/*σ*] − Φ[(*μ*_*cB*_ − *μ*_*nB*_)/*σ*].

For a paired comparison of the AUCs of the two screening tests, we test the hypothesis *H*_0_ : (*μ*_*cA*_ − *μ*_*nA*_) − (*μ*_*cB*_ − *μ*_*nB*_) = (*μ*_*cA*_ − *μ*_*cB*_) − (*μ*_*nA*_ − *μ*_*nB*_) = 0 against *H*_*A*_ : ¬ *H*_0_. If *H*_0_ holds, the AUCs, and hence the diagnostic accuracies of the two screening tests, are equal. To test *H*_0_, we fit a general linear univariate model with the difference in the screening test scores as the outcome. The approach was inspired by the work of Demler et al. [21]. We assume that the difference between screening test scores is Gaussian and that the observations on different participants are independent.

The general linear univariate model for the final data set can be written as ***Y***_+_ = *X*_+_***β*** + *ϵ*_+_, where ***Y***_+_ is an *N*_+_ × 1 matrix containing the difference in the screening test scores for each individual, [*y*_*idA*_ − *y*_*idB*_]^'^, *X*_+_ is an *N*_+_ × 2 design matrix that identifies disease status, ***β*** is a 2 × 1 matrix of mean differences [*μ*_*cA*_ − *μ*_*cB*_*μ*_*nA*_ − *μ*_*nB*_]^'^, and *ϵ*_+_ is the *N*_+_ × 1 matrix of errors. We test *H*_0_ by writing the contrast matrix ***C*** = [1–1], forming ***θ*** = ***Cβ***, and using an *F* statistic ([22], p. 51, Equation 2.32). The final *F* statistic used in our adapted internal pilot design is written as *F*_+_.

#### Sample size re-estimation for an internal pilot with unknown disease prevalence

The initial sample size is calculated as in Muller et al. [23]. For that calculation, the study investigator will specify *σ*_0_^2^ and *β*_0_. Ideally, speculated values will be based on data from previous studies, closely related published results, or clinical experience.

After the internal pilot, the final sample size can be re-calculated using the following iterative algorithm. The goal of the algorithm is to find *N*_+_, where the power of the study is equal to *P*_*t*_, the target power. First, check to see if the pilot data includes either all cases or all non-cases. If so, set *N*_+_ = *n*_0_. Otherwise, calculate the final sample size as follows. With *n*_*c*1_ and *n*_*n*1_ as observed in the initial pilot, define *κ* to be the greatest common factor of *n*_*c*1_ and *n*_*n*1_. Let *D* = *n*_*c*1_/*κ*, *E* = *n*_*n*1_/*κ*, and *R* = (*D* + *E*).

Speculate that ***X***_+_ will take the form ***X***_+_ = Es(***X***) ⊗ 1_*m*_, where Es(***X***) is an (*R* × 2) matrix such that

1 $$ \mathrm{E}\mathrm{s}\left(\boldsymbol{X}\right)=\left[\begin{array}{cc}\hfill {1}_D\hfill & \hfill {0}_D\hfill \\ {}\hfill {0}_E\hfill & \hfill {1}_E\hfill \end{array}\right], $$

and *m* is a positive integer chosen so that *N*_+_ = *mR* ≥ *n*_1_.

Calculate the power as 1 − Pr[*F*_+_ ≤ *f*_crit_] [23], where *f*_crit_ = *F*_*F*_^− 1^[(1 − *α*_*t*_); 1, *N*_+_ − 2] and *F*_+_ has a non-central *F* distribution with 1 numerator degrees of freedom, denominator degrees of freedom *N*_+_ − 2, and non-centrality parameter ${\omega}_{+}={\delta}_{+}/{\widehat{\sigma}}_1^2$, where ${\delta}_{+}={\left(\boldsymbol{\theta} -{\theta}_0\right)}^{\mathit{\hbox{'}}}{\left[\boldsymbol{C}{\left({\boldsymbol{X}}_{+}^{{}^{\mathit{\hbox{'}}}}{\boldsymbol{X}}_{+}\right)}^{-}{\boldsymbol{C}}^{\mathit{\hbox{'}}}\right]}^{-1}\left(\boldsymbol{\theta} -{\theta}_0\right)$.

Sequentially increment or decrement *m* until the power of the experiment meets or exceeds *P*_*t*_, at *m* = *m*_*t*_. Set the final sample size to be *N*_+_ = *m*_*t*_*R*, unless *N*_+_ ≥ *N*_*max*_ or *N*_+_ ≤ *N*_*min*_. If *N*_+_ ≥ *N*_*max*_ then set *N*_+_ = *N*_*max*_. If *N*_+_ ≤ *N*_*min*_ then set *N*_+_ = *N*_*min*_. Finally, calculate *N*_2_ as *N*_2_ = *N*_+_ − *n*_1_.

### Simulation studies

#### Verification of unconditional power

We conducted a simulation study designed to verify the result of Equation (10) below. Simulation study parameters came from modifying an example presented in Kairalla et al. [24]. Kairalla et al. [24] modified a balanced example in Wittes and Brittain [8] so that the numbers of cases to non-cases were unequal. Kairalla et al. then assumed a fixed case mixture throughout the study. We, in turn, modified the example in [24] by allowing the ratio of cases to non-cases to vary randomly.

Initial parameters were set at: ***C*** = [1–1], *P*_*t*_ = 0.90, *α*_*t*_ = 0.05, *β* = [1 0]^'^, and *σ*_0_^2^ = 2. The resulting initial sample size was *n*_0_ = 96 participants. With *λ* = 0.5, the pilot sample was fixed at *n*_1_ = 48. The true rate of disease was set at *π* = 1/3. The parameter *y* ranged between 0.5 to 2 by 0.25 while *γ*_*π*_ was fixed at 1. Under the alternative hypothesis, the bivariate normal parameters were set at *μ*_*c*1_ = 3, *μ*_*c*2_ = 4, *ρ*_*c*12_ = 0, *μ*_*n*1_ = 0, *μ*_*n*2_ = 0, *ρ*_*n*12_ = 0, and *σ*_*c*_^2^ = *σ*_*n*_^2^ = 1. To calculate Type I error under *H*_0_, the bivariate normal parameters were set at *μ*_*c*1_ = 3, *μ*_*c*2_ = 3, *ρ*_*c*12_ = 0, *μ*_*n*1_ = 0, *μ*_*n*2_ = 0, *ρ*_*n*12_ = 0, and *σ*_*c*_^2^ = *σ*_*n*_^2^ = 1. The distributional parameters under the null correspond to an AUC of 0.983 and a difference in AUC of 0.015 under the alternative. All programs were written in version 9.3 of SAS/IML® software [25] and are available upon request. The empirical power was calculated as the proportion of times the null hypothesis was rejected. The experiment was repeated 10,000 times. The maximum absolute deviation (MAD) was calculated as the maximum absolute difference between the empirical estimates and the theoretical value. Using a normal approximation to the distribution of a proportion, the half-width of the 95 % CI for a target power of 0.90 is 0.0053.

#### Assessment of Type I error rate inflation

We conducted a simulation study to assess the magnitude of the Type I error rate inflation for a variety of experimental conditions. The Type I error rate was simulated for a prospective cancer screening trial with an internal pilot design. The disease prevalence and variance were either correctly or incorrectly specified and then re-estimated using pilot data. The hypothesis test was conducted using either an adjusted or unadjusted critical value.

The empirical Type I error was calculated for 648 different scenarios. The null hypothesis was that there was no difference in the diagnostic accuracy of the screening tests. For each scenario, we simulated 10,000 replicate data sets, conducted the hypothesis test, formed the *P*-value, and decided whether to accept or reject the null hypothesis at the *α*_*t*_ = 0.05 level. The number of replicates was chosen so that the 95 % confidence interval of the proportion was no more than 0.005. The empirical Type I error was calculated as the proportion of replicates where the null hypothesis was rejected. For some scenarios, the study population was composed of either all cases or all non-cases. For all such scenarios, we considered there to be insufficient evidence to reject the null hypothesis.

The 648 different scenarios came from a range of parameter values. Parameters of the bivariate normal distributions for the cases and non-cases were fixed at ***μ***_*n*_ ∈ {[0 0]^'^}, ***μ***_*c*_ ∈ {[0.2 0.2]^'^, [0.5 0.5]^'^}, *σ*_0_^2^ ∈ {0.34}, and *ρ*_*n*_ = *ρ*_*c*_ = 0.5. This corresponded to a difference in the AUCs of test *A* and test *B* of 0.05 or 0.1, respectively. The proportion of the initial sample size used for the internal pilot was in the range of *λ* ∈ {0.25, 0.5, 0.75}. We varied target power, *P*_*t*_ ∈ {0.80, 0.90}, the ratio of the true variance to the initial variance estimate, *γ* ∈ {0.5, 1, 1.5}, and the ratio of the true population disease prevalence to the initial prevalence estimate, *γ*_*π*_ ∈ {0.1, 1, 1.9}. The initial prevalence estimate was fixed at *π*_0_ = 0.5, corresponding to a balanced study design.

#### Validation of Type I error control

We compared our adjusted method to an unadjusted internal pilot approach for a scenario where significant Type I error inflation occurred. The parameters that defined the scenario were ***μ***_*n*_ = {[0 0]^'^}, ***μ***_*c*_ = {[0.3 0.92]^'^}, *σ*_0_^2^ ∈ {0.34}, *ρ*_*n*_ = *ρ*_*c*_ = 0.5, *π* = 0.5, and *λ* ∈ {0.5}. The parameters correspond to an AUC of 0.64 for test *A* and an AUC of 0.87 for test *B*. We varied *γ* between 0.25 and 4. With *P*_*t*_ = 0.90 and *α* = 0.05, the initial sample size was 42. The adjusted method was applied to each of three possible prevalence misspecification scenarios with *γ*_*π*_ ∈ {0.1, 1, 1.9}.

## Results

### Type I error rate control

#### Overview

In general, internal pilot studies can inflate Type I error rate [14]. Here, we describe a method to bound Type I error rate in internal pilot studies where both the variance of the outcome and the disease prevalence are re-estimated in the internal pilot step. First, we give the unconditional power and hence the Type I error for the *F* test statistic. We uncondition over all possible realizations of *N*_1_, *N*_*c*1_, *N*_*c*2_, and *N*_2_. After demonstrating that the Type I error rate takes on a maximum value across a specified range of *γ* and *γ*_*π*_, we describe a method for identifying the values of *γ* and *γ*_*π*_ at which the maximum occurs. We choose a critical value for the final hypothesis test so that the maximum Type I error rate is bounded.

#### Unconditional Type I error

We derive the distribution of the *F*_+_ statistic under *H*_0_ and *H*_*A*_. Under *H*_0_, the formulae give an unconditional Type I error. Under *H*_*A*_, the formulae give unconditional power. Because both the variance and the disease prevalence are re-estimated, the test statistic is a function of the pilot sample size and the final sample size. Derivation of the distribution of the test statistic requires obtaining three results:

1. The distributions of *N*_1_, *N*_*c*1_, *N*_*c*2_, *N*_2_, and *N*_+_.
2. The distribution of *F*_+_ conditional on *N*_1_, *N*_*c*1_, *N*_*c*2_, *N*_2_, and *N*_+_.
3. The unconditional Type I error and power of the *F*_+_ test statistic.

Under the Type I error rate control subsection each of the three afore mentioned results are presented. Throughout the this subsection we find it useful to use functional notation to emphasize the dependence of variables on *N*_1_, *N*_*c*1_, *N*_*c*2_, *N*_2_, and *σ*_1_^2^. For example, we write *N*_2_(*σ*_1_^2^, *N*_*c*1_, *N*_1_) to indicate that the additional sample size is a function of the pilot variance and the pilot case mixture.

#### Distributions of , *N*_1_, *N*_c1_, *N*_c2_, and *N*_2_

The number of participants in the pilot sample is fixed by study design: *n*_1_ = *λn*_0_. Assuming a true disease prevalence of *π*, *N*_*c*1_ ∼ Binomial(*n*_1_, *π*) and *N*_*c*2_ ∼ Binomial(*N*_2_, *π*). The random variables ${\widehat{\sigma}}_1^2$ and *N*_*c*1_ are distributed independently. Summing over all possible values of *n*_*c*1_, the unconditional probability mass distribution of the additional sample is:

2 $$ \begin{array}{cc} \Pr \left\{{N}_{+}={n}_{+}\right\}& =\sum_{n_{c1i}=0}^{n_1} \Pr \left\{{N}_{+}={n}_{+}|{N}_{c1i}={n}_{c1i}\right\}\times \Pr \left\{{N}_{c1i}={n}_{c1i}\right\}\\ {}=\sum_{n_{c1i}=0}^{n_1}\left( \Pr \left\{{N}_{+}\le {n}_{+}+1|{n}_{c1i}\right\}- \Pr \left\{{N}_{+}\le {n}_{+}|{n}_{c1i}\right\}\right)\\ {}\kern4em \times \Pr \left\{{N}_{c1i}={n}_{c1i}\right\},\end{array} $$

where the first line extends Equation 18 of [9], and the second line follows from the law of total probability. The conditional probability mass function of *N*_+_ is calculated by extending Equation 17 of [9] as follows:

3 $$ \Pr \left\{{N}_{+}\le {n}_{+}\Big|{n}_{c1i}\right\}= \Pr \left\{{\chi}^2\left({n}_1-2\right)\le \frac{\left({n}_1-2\right)}{\sigma^2}\;\frac{\delta_{+}}{\omega_{+}}\;\Big|\;{N}_{c1i}={n}_{c1i}\right\}. $$

Note that since *N*_2_ = *N*_+_ − *n*_1_,

4 $$ \Pr \left\{{N}_{+}\le {n}_{+}\Big|{n}_{c1i}\right\}= \Pr \left\{{N}_2\le {n}_2\Big|{n}_{c1i}\right\}. $$

#### Power of the final hypothesis test conditional on N_1_, N_c1_, N_c2_, N_2_, and N_+_

We show the dependence of the power on *N*_1_, *N*_*c*1_, *N*_*c*2_, and *N*_2_.

The additional sample size *N*_2_ is a function of ${\widehat{\sigma}}_1^2$ and *N*_*c*1_. Since the power function is strictly monotone increasing, for fixed values of ${\widehat{\sigma}}_1^2$, *n*_1_, and *n*_*c*1_, there exists one and only one *N*_2_ = *n*_2_. However, for a fixed *n*_1_ and *n*_*c*1_, there exist infinitely many ${\widehat{\sigma}}_1^2$, all of which would yield the same final sample size.

Let *q*_1_(*n*_2_, *n*_*c*1_) and *q*_2_(*n*_2_, *n*_*c*1_) represent the smallest and the largest value of ${\widehat{\sigma}}_1^2$ that would lead to the additional sample size *n*_2_ for a fixed *n*_1_ and *n*_*c*1_. Let *q*(*n*_2_, *n*_*c*1_) be the value of ${\widehat{\sigma}}_1^2$ that falls in the interval (*q*_1_(*n*_2_, *n*_*c*1_), *q*_2_(*n*_2_, *n*_*c*1_)].

We can express the approximate power of the *F*_+_ test statistic for a value *f*(*n*_2_, *n*_*c*2_, *n*_*c*1_) as a function of *n*_2_, *n*_*c*2_, and *n*_*c*1_. Let *I*(*n*_2_, *n*_*c*2_, *n*_*c*1_) represent the probability of rejecting *H*_0_ when the alternative is true, conditional on *n*_*c*2_, *n*_*c*1_ and the value *q*(*n*_2_, *n*_*c*1_). Then

5 $$ \begin{array}{cc}I\left({n}_2,\ {n}_{c2},\ {n}_{c1}\right)& =1- \Pr \left\{{F}_{+}\le f\left({n}_2,\ {n}_{c2},\ {n}_{c1}\right)\Big|q\left({n}_2,\ {n}_{c1}\right),\ {n}_{c2},\ {n}_{c1}\right\}\\ {}=1- \Pr \Big\{c\left({n}_2,\ {n}_{c2},\ {n}_{c1}\right)\cdot {\chi}^2\left[a,{\omega}_{+}\left({n}_1+{n}_2,\ {n}_{c2}+{n}_{c1}\right)\right]\\ {}\kern2em -{\chi}^2\left({n}_2\right)\le q\left({n}_2,\ {n}_{c1}\right)\left|q\left({n}_2,\ {n}_{c1}\right),{n}_{c2},\ {n}_{c1}\right\},\end{array} $$

where *ν*_+_ = *N*_+_ − 2, *c*(*n*_2_, *n*_*c*2_, *n*_*c*1_) = *ν*_+_/[2*f*(*n*_2_, *n*_*c*2_, *n*_*c*1_)] with *χ*^2^[*a*, *ω*_+_(*n*_1_ + *n*_2_, *n*_*c*2_ + *n*_*c*1_)] denoting a non-central *χ*^2^ with *a* degrees of freedom and a non-centrality parameter of *ω*_+_(*n*_1_ + *n*_2_, *n*_*c*2_ + *n*_*c*1_). Equation (5) follows from the proof in the Appendix of Coffey and Muller [9].

#### Expected power of the **F** test statistic unconditioned from N_1_, N_c1_, N_c2_, N_2_, and N_+_

We uncondition Equation (5) from *N*_*c*1_, *q*(*n*_2_, *n*_*c*1_), *N*_*c*2_, and *N*_2_. Using the law of total probability, the unconditional power is

6 $$ I\left({n}_2,\ {n}_{c2}\right)=1-\sum_{n_{c1i}=0}^{n_1}I\left({n}_2,\ {n}_{c2},\ {n}_{c1i}\right)\times Pr\left[{N}_2={n}_2\Big|{n}_{c1i}\right]\times Pr\left[{N}_{c1}={n}_{c1i}\right]. $$

Substituting Equation (6) into Equation (5) gives

7 $$ \begin{array}{ll}I\left({n}_2,\ {n}_{c2}\right)& =1-\sum_{n_{c1i}=0}^{n_1} \Pr \left\{Q\left({n}_2,\ {n}_{c2},\ {n}_{c1i}\right)\le q\left({n}_2\Big|{n}_{c1i}\right)\Big|q\left({n}_2\Big|{n}_{c1i}\right),\ {n}_{c2},\ {n}_{c1i}\right\}\kern1em \\ {}\kern1em & \kern4em \times Pr\left[{N}_2={n}_2\Big|{n}_{c1i}\right]\times Pr\left[{N}_{c1}={n}_{c1i}\right].\kern1em \end{array} $$

Unconditioning the power from *N*_*c*2_, we obtain

8 $$ I\left({n}_2\right)=1-\sum_{n_{c2i}=0}^{n_2}I\left({n}_2,\ {n}_{c2i}\right)\times Pr\left[{N}_{c2}={n}_{c2i}\Big|{n}_2\right], $$

leading to

9 $$ \begin{array}{cc}I\left({n}_2\right)& =1-\sum_{n_{c2i}=0}^{n_2}\Big(\sum_{n_{c1i}=0}^{n_1} Pr\left\{Q\left({n}_2,\ {n}_{c2},\ {n}_{c1i}\right)\le q\left({n}_2|{n}_{c1i}\right)\Big|q\left({n}_2|{n}_{c1i}\right),\ {n}_{c2},\kern0.5em {n}_{c1i}\right\}\\ {}\kern4em \times Pr\left[{N}_2={n}_2|{n}_{c1i}\right]\times Pr\left[{N}_{c1}={n}_{c1i}\right]\Big)\times Pr\left[{N}_{c2}={n}_{c2i}|{n}_2\right]\\ {}\kern4em \\ {}=1-\sum_{n_{c2i}=0}^{n_2}\Big(\sum_{n_{c1i}=0}^{n_1}{\int}_{q_1\left({n}_2,\kern0.5em {n}_{c1}\right)}^{q_2\left({n}_2,\kern0.5em {n}_{c1}\right)} Pr\left\{Q\left({n}_2,\kern0.5em c\left({n}_2,\ {n}_{c2i},\kern0.5em {n}_{c1i}\right),\ \delta \left({n}_{c2i},\kern0.5em {n}_{c1i}\right)\right)\le t\right\}\\ {}\kern4em \times \frac{f_{\chi^2}\left(t,{\nu}_1\right)}{Pr\left\{{N}_2={n}_2|{n}_{c1i}\right\}}dt\times Pr\left[{N}_2={n}_2|{n}_{c1i}\right]\times Pr\left[{N}_{c1}={n}_{c1i}\right]\Big)\\ {}\kern4em \times Pr\left[{N}_{c2}={n}_{c2i}|{n}_2\right],\end{array} $$

with ${f}_{\chi^2}\left(t,\;{\nu}_1\right)$ defined in Johnson et al. [26]. The distributional results of Coffey et al. [19] hold, conditional on fixed values of *N*_1_, *N*_*c*1_, and *N*_*c*2_. The expected power is given by

10 $$ \begin{array}{c} Pr\left\{{F}_{+}\left({N}_{+}, {N}_{c+}, {N}_{c1}\right)\le f\left({N}_{+}, {N}_{c+}, {N}_{c1}\right)\right\}\\  =1-\sum\limits_{n_{+i}={n}_1}^{n_1+{n}_2}\ \sum\limits_{n_{+ci}={n}_{c1}}^{n_{c1}+{n}_{c2}}\ \sum\limits_{n_{c1i}=0}^{n_1}{\int}_{q_{1\left({n}_{2i},\ {n}_{c1i}\right)+}}^{\infty }{F}_{\chi^2}\left[\frac{z}{c{\left({n}_{c+i}\right)}_{+}};2,\frac{\delta {\left({n}_{c+i}\right)}_{+}}{\gamma {\sigma}_0^2}\right]{f}_{\chi^2}\left(z;{\nu}_{+}\right)\\   \times {F}_{\beta}\left(\frac{q_{2\left({n}_{2i},\ {n}_{c1i}\right)}}{z};\frac{\nu_1}{2},\frac{n_{2i}}{2}\right)-{F}_{\beta}\left(\frac{q_{1\left({n}_{2i},\ {n}_{c1i}\right)+}}{z};\frac{\nu_1}{2},\frac{n_{2i}}{2}\right)dz \\  \times Pr\left[{N}_{c1}={n}_{c1i}\right]\times Pr\left[{N}_{c2}={n}_{c2i}\Big|{n}_{2i}\right], \end{array} $$

where *F*_+_(*N*_+_, *N*_*c* +_, *N*_*c*1_) is the final test statistic, *f*(*N*_+_, *N*_*c* +_, *N*_*c*1_) is an observed value, ${F}_{\chi^2}$ is the cumulative distribution function of a non-central *χ*^2^ [27], *F*_*β*_ is the cumulative distribution function of a beta (one) distributed random variable [27], *ν*_1_ = *n*_1_ − 2, and the bounds of the integration depend on *n*_*c*1_ and *n*_2_. The Type I error can be calculated from Equation (10) when the null hypothesis is true. Notice that when the null hypothesis is true, the *χ*^2^ distribution in Equation (10) becomes a central *χ*^2^.

#### Bounding Type I error

There exists a maximum Type I error across a specified range of *γ* and *γ*_*π*_. Let *α*^*max*^ be the global maximum Type I error. Power for a study design is maximized when the ratio of the number of study participants with disease to the number of study participants without disease is one-to-one. Thus, *α*^*max*^ must occur for *γ*_*π*_ = 1. The problem of showing that there is a maximum then reduces to showing that there exists a maximum with respect to *γ* for *γ*_*π*_ = 1. Coffey and Muller [12] provide evidence to support this assertion.

We propose the following method to find the *γ* = γ* and *γ*_*π*_ = *γ*_*π*_^*b*^ for which the maximum Type I error occurs:

9. First, fix a range for *γ*∈ [*a*, *b*] and *γ*_*π*_∈ [*c*, *d*] a priori, based on the previous literature.
10. Find the value of *γ*_*π*_ = *γ*_*π*_^*b*^ that results in a study design with a permissible prevalence value that is closest to a one-to-one ratio of cases to non-cases (that is, the value closest to 1 ∈ [*c*, *d*]).
11. Finally, for a fixed *γ*_*π*_^*b*^, find the value of *γ* = γ* that yields the maximum Type I error inflation, using Equation (10) and a golden section search algorithm [28].

The maximum Type I error is bounded by identifying an adjusted critical value for the final test statistic. For *γ* = γ* and *γ*_*π*_ = *γ*_*π*_^*b*^ we use a bisection search algorithm to find α* so that under *H*_0_, Pr{*F*_+_(*N*_+_, *N*_*c* +_, *N*_*c*1_) ≤ *f*_adj_} = *α*_*t*_, where *f*_adj_ = *F*_*F*_^− 1^[(1 − α*); 1, *N*_+_ − 2].

### Simulation studies results

#### Verification of unconditional power

The simulation study suggested that for the parameters chosen, Equation (10) provides a good estimate of unconditional power. The MAD between predicted empirical power and theoretical power always fell within the 95 % confidence interval (Tables 1 and 2). The half-width of the 95 % CI for a target Type I error of 0.05 is 0.0043.

**Table 1:** Empirical versus theoretical power by variance misspecification

*γ*	Empirical power	Theoretical power	Absolute deviation
0.5	0.9945	0.995	0.0005
0.75	0.9646	0.964	0.0006
1	0.9369	0.934	0.0029
1.25	0.9168	0.922	0.0052
1.5	0.9144	0.916	0.0016
1.75	0.9137	0.913	0.0007
2	0.9136	0.910	0.0036

**Table 2:** Empirical versus theoretical Type I error by variance misspecification

*γ*	Empirical type I error	Theoretical type I error	Absolute deviation
0.5	0.0505	0.053	0.0025
0.75	0.0487	0.053	0.0043
1	0.0496	0.052	0.0024
1.25	0.0505	0.052	0.0015
1.5	0.0514	0.052	0.0006
1.75	0.0478	0.051	0.0032
2	0.0507	0.051	0.0003

#### Assessment of Type I error rate inflation

**Fig. 2:**
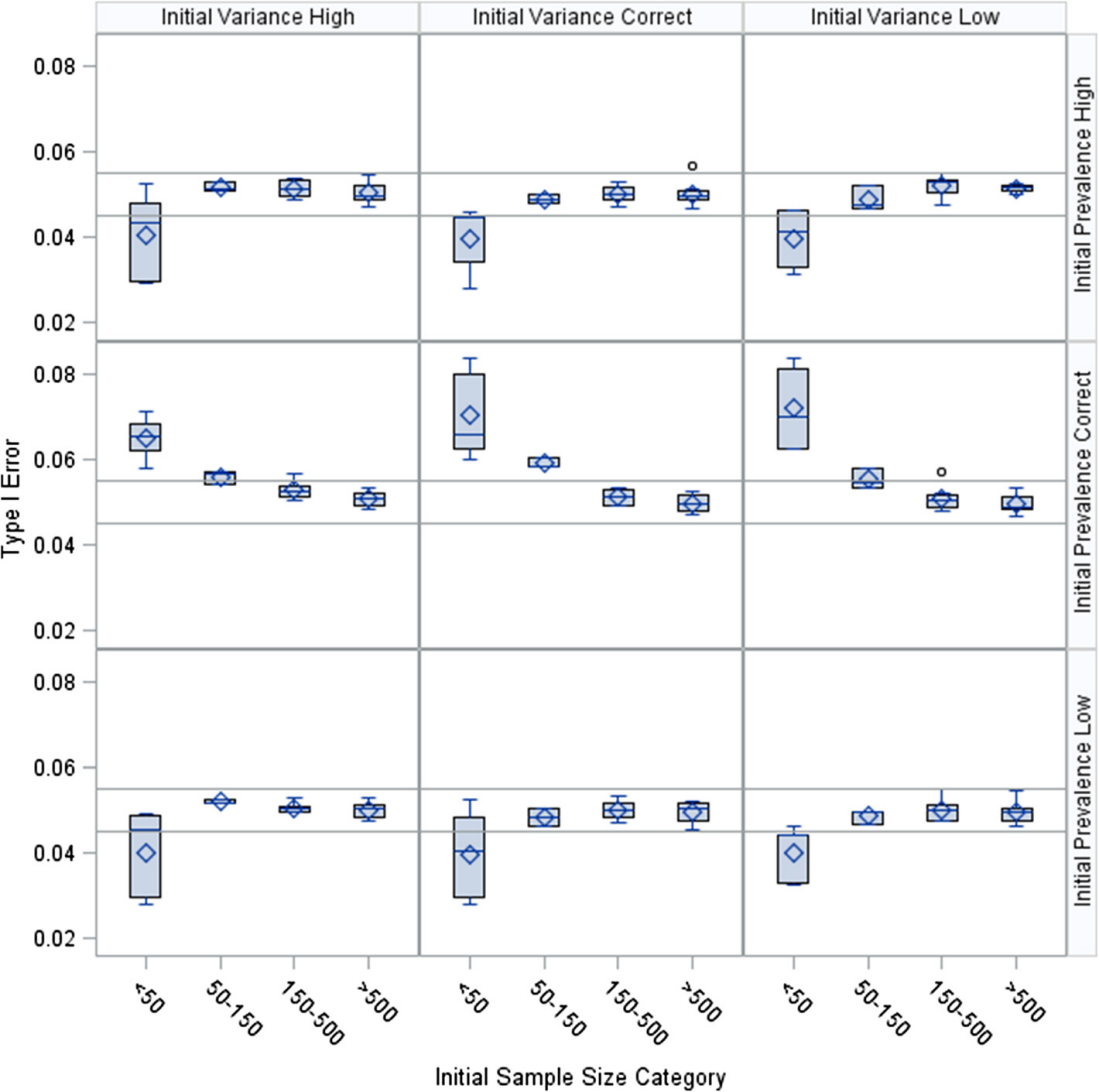
Type I error rate by scenario with the pilot study size at 25 % of initial sample size estimate

**Fig. 3:**
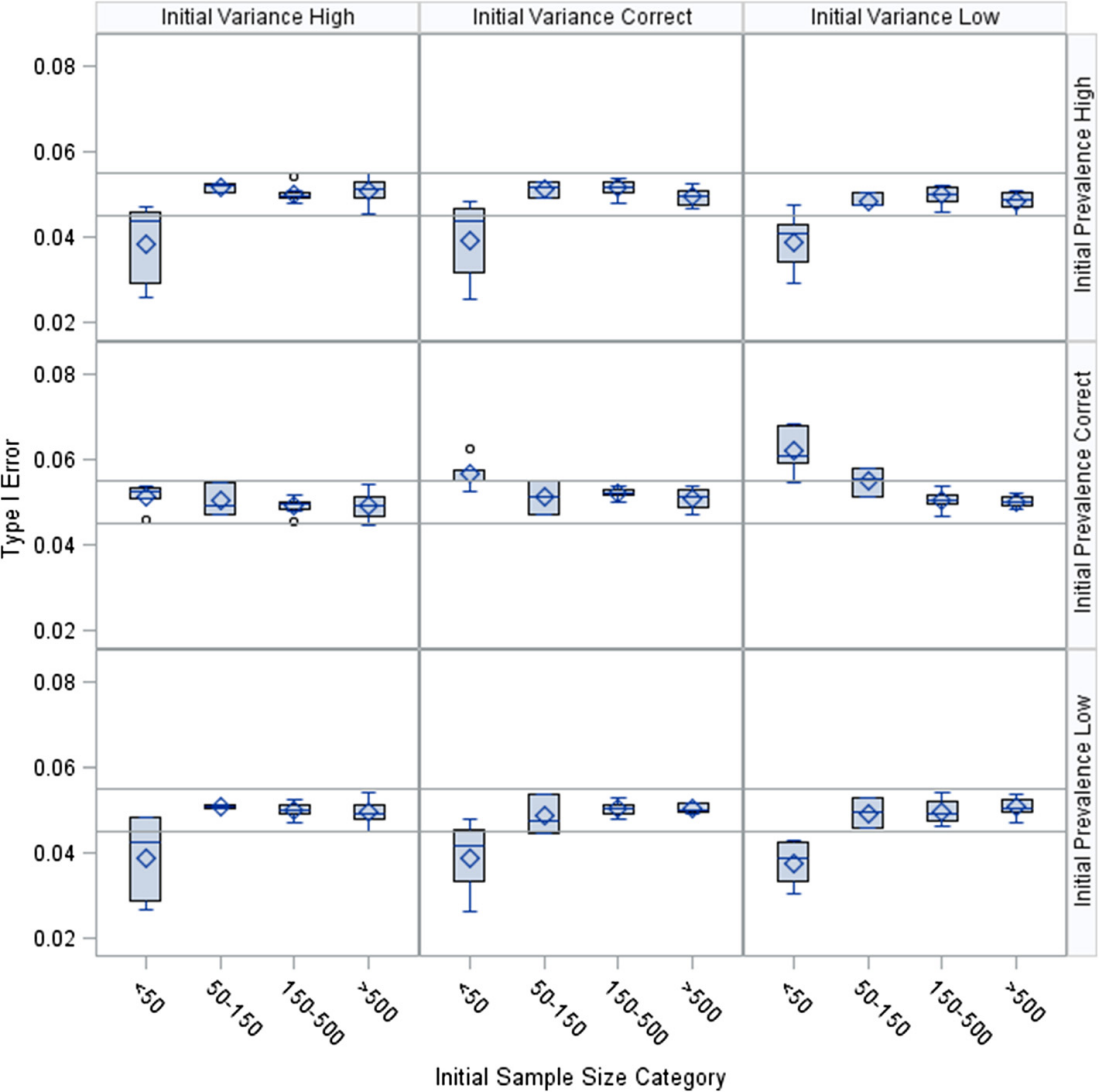
Type I error rate by scenario with the pilot study size at 50 % of initial sample size estimate

**Fig. 4:**
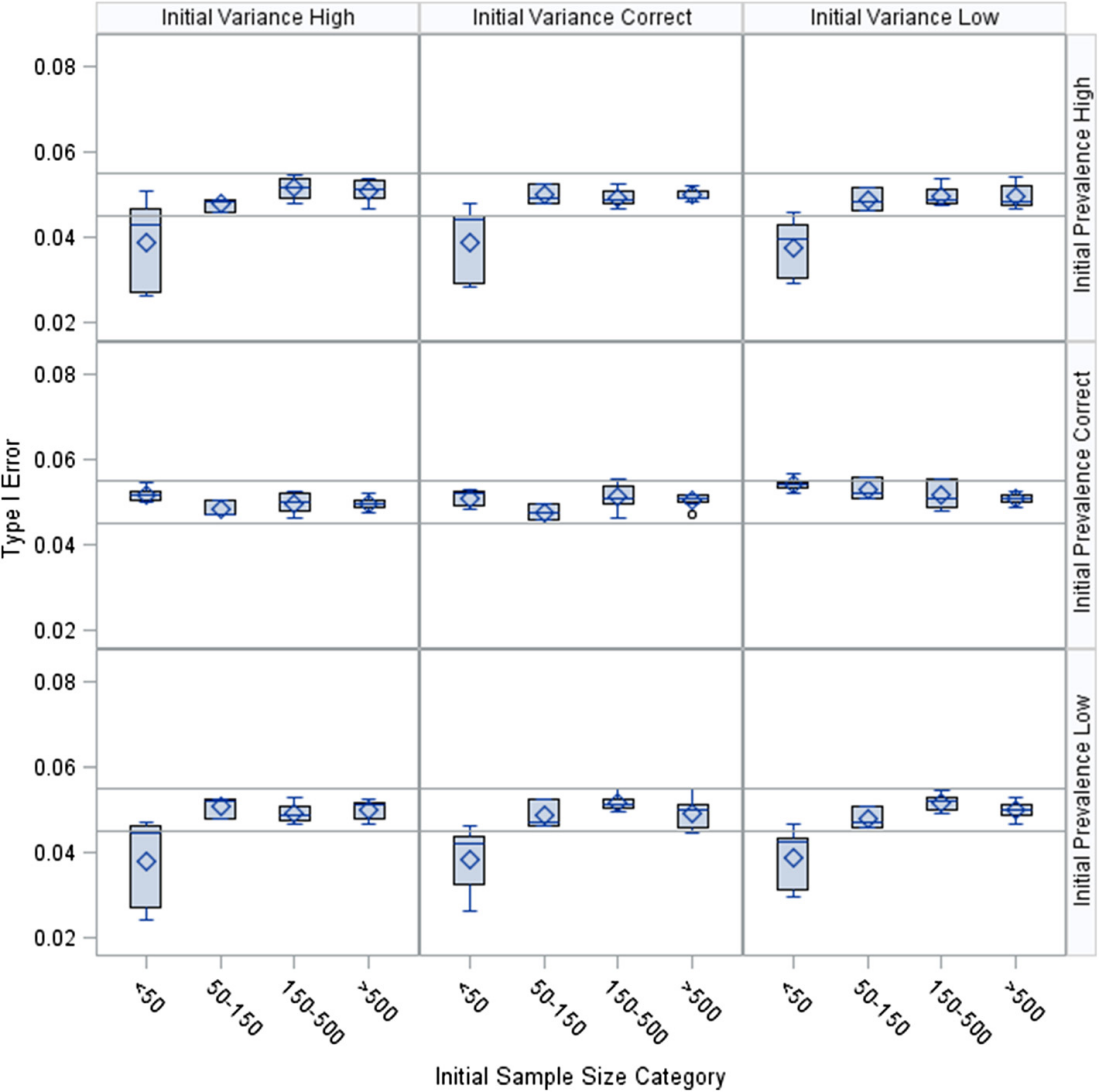
Type I error rate by scenario with the pilot study size at 75 % of initial sample size estimate

Results from the simulation are presented in Figs. 2, 3, and 4. Overall, the Type I error rate was inflated when the initial sample size was smaller than 50 and the initial prevalence estimate was correct. As the fraction of the initial sample size estimate used in the pilot study increased, the inflation grew smaller. The initial sample sizes for all 648 scenarios ranged from 12 to 2,028 participants, with an interquartile range of 61 to 635 participants. The median observed Type I error was 0.0495, with a minimum of 0.0244, a maximum of 0.0839, and an interquartile range of 0.0479 to 0.0521.

The figures suggest that no Type I error adjustment is needed when the sample size is large. This observation is consistent with the results from Wu et al. [11]. The results from the simulation study by Wu et al. [11] correspond to the subset of results in Figs. 2, 3, and 4 with *γ* = 1 and *γ*_*π*_ = 1. However, Wu et al. [11] did not consider cases with small initial sample sizes, and thus did not observe the Type I error rate inflation shown in our results. In our first example, we present an application with a large sample size where no adjustment is needed to bound the Type I error rate.

#### Validation of Type I error control

**Fig. 5:**
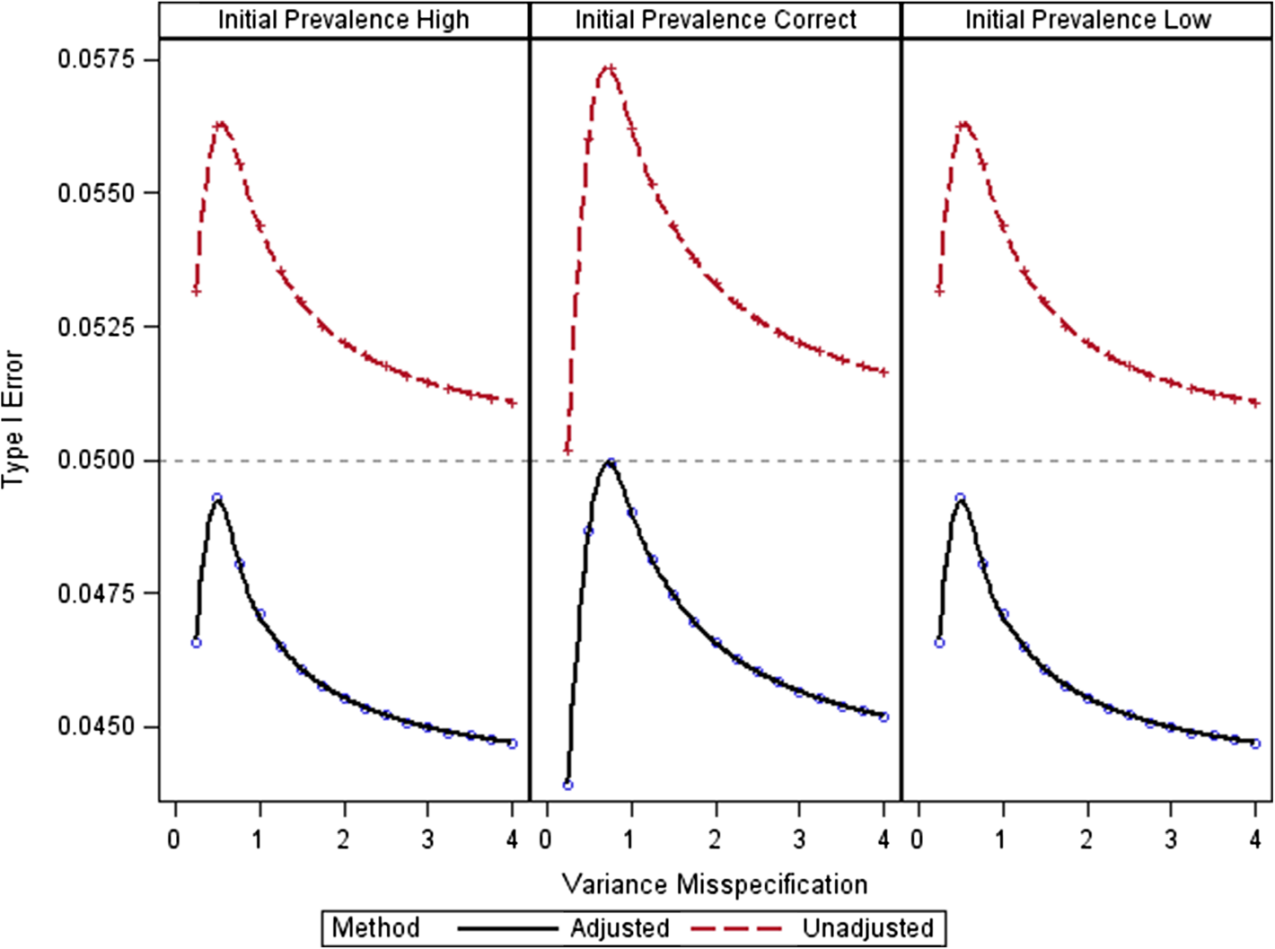
Bounded Type I error

Results from the Type I error control simulation appear in Fig. 5, which shows a comparison of the Type I error inflation for the adjusted and unadjusted methods. The figure plots Type I error rate as a function of *γ*, cross-classified by *γ*_*π*_ for the two methods. Figure 5 shows that the adjusted method controls the Type I error rate in small samples. The maximum possible Type I error occurred with *γ*_*π*_^*b*^ = 1, γ* = 0.8541 for a Type I error of 0.0564. The adjusted Type I error rate was α* = 0.0438. Note that *f*_*adj*_ is only assigned a value after the pilot sample is collected and *N*_+_ = *n*_+_ is re-estimated.

### Applications

#### Example 1: A large oral cancer screening trial where no adjustment is needed

One implication of this study is that internal pilot designs often require no penalty for re-estimating both outcome variance and disease prevalence. In addition, the internal pilot design ensures that researchers will have sufficient power.

Recall the study by Lingen et al. discussed in the Background section. One aim of the study was to compare the diagnostic accuracy of a combined modality involving both visual and tactile oral exam with VELscope® [29]. The investigators wished to detect oral pre-malignancy and malignancy. There was substantial uncertainty about the rate of oral pre-malignancy and malignancy in the target population. The rate of suspicious lesions varies widely in Western populations, ranging from 0.2 % to 16.7 % [5]. Further, the variance of scores for visual and tactile oral exam and for examinations with VELscope was largely unknown. The uncertainty made an internal pilot design attractive.

One critical step for designing an internal pilot study is choosing *N*_min_ and *N*_max_. The investigators wished to estimate a confidence interval for the percentage of oral lesions that were benign. To ensure that the confidence interval had a half-width of no more than 0.1 %, the investigators had to make sure that the entire study enrolled at least 96 people with lesions. If the rate of suspicious lesions was about 12.1 %, the minimum sample size could be no less than 800. The upper bound on sample size was fixed by monetary constraints. Previous experience had shown that a sample size of more than 30,000 was fiscally unfeasible. This set *N*_max_ at 30,000.

The initial power calculation was based on plausible values from the literature. A conservative estimate for the AUC for visual and tactile oral exam is 0.60. A clinically interesting difference between AUCs is 0.06. This corresponds to ***μ***_*n*_ ∈ {[0 0]^'^}, ***μ***_*c*_ ∈ {[0.359 0.584]^'^}, *σ* = 1, and *ρ*_*n*_ = *ρ*_*c*_ = 0. Assuming that the rate of suspicious lesions in the population is 12.1 %, the initial sample size needed for 95 % power is 2,156 non-cases and 294 cases for a total sample size of 2,450.

The final sample size that would be needed for the study would depend on results from the internal pilot. The results presented in the Type I error control validation indicate that Type I error inflation would not be a problem for a study designed with an initial sample size of 2,450. Thus, the final hypothesis test could be carried out with *α* set to 0.05.

#### Example 2: A small oral cancer screening trial where adjustment prevents Type I error inflation

A second implication of this manuscript is that internal pilot designs with small sample size require an adjustment to prevent Type I error inflation. Small sample sizes often occur because of biological constraints. For example, Wong et al. [30] are currently recruiting for an oral cancer screening trial in people with Fanconi anemia. Fanconi anemia is a rare genetic disease that occurs in roughly 1 in 131,000 people in the United States. People with Fanconi anemia are at increased risk for oral cancer, although the magnitude of the risk is unknown. The prevalence of oral squamous cell carcinoma could be as high as 100 % or as low as 3 % [31, 32].

Because the study is still in progress, the design has not yet been published. To illustrate the results of our manuscript, we show how an internal pilot trial might be used to compare the diagnostic accuracy of two assays for IL-8 for the prediction of oral cancer. In people with Fanconi anemia, IL-8 is a useful biomarker for screening for oral cancer [33, 34].

Consider a trial in which people with Fanconi anemia are given two salivary assays: a salivary bead-based assay for IL-8, and an enzyme-linked immunosorbent assay (ELISA). The diagnostic accuracy (AUC) of the ELISA and the salivary bead-based assay is 0.85 and 0.94, respectively [34, 35]. The target power is set to 0.80. A clinically interesting difference in diagnostic accuracy is a difference between AUCs of 0.09. The target Type I error rate is 0.05. Means and variances of both ELISA and a salivary bead-based assay are available in the literature [34, 35], with ***μ***_*n*_ ∈ {[759.4 759.4]^'^}, ***μ***_*c*_ ∈ {[3347.7 4700.0]^'^}, and *σ*_*nA*_ = *σ*_*nB*_ = *σ*_*cA*_ = *σ*_*cB*_ = 3328174.5. Modest correlation is set at *ρ*_*n*_ = *ρ*_*c*_ = 0.5.

If half the people in the study have oral cancer, the initial sample size required is 84 participants. Thus, the study could be subject to Type I error inflation. If we re-estimate the sample size after the first 42 participants have been collected, the study could have a Type I error rate inflated to 0.054. This inflation occurs at *γ*_*π*_^*b*^ = 1 and γ* = 0.7254. This is an 8 % inflation from the target Type I error rate of 0.05. Adjusting gives an adjusted alpha level of *α*_adj_ = 0.0463. The adjusted critical value can be calculated as *f*_adj_ = *F*_*F*_^− 1^[(1 − α*); 1, *N*_+_ − 2]. Recall that the actual adjusted critical value will depend on the final sample size calculated after the internal pilot is observed. For example, if *n*_+_ = 100, then *f*_adj_ = 4.07. Thus with *n*_+_ = 100, any observed test statistic larger than 4.07 should be rejected.

## Discussion

In this manuscript, we describe an internal pilot approach for cancer screening trials when the disease prevalence is unknown. We demonstrated that conducting an internal pilot study without adjusting the critical value caused Type I error rate inflation in small (*N* <50) samples, but not in large samples. We also demonstrated that our adjusted method controlled Type I error rate in small samples.

The approach has both strengths and limitations. A strength is that the method allows investigators to obtain expected power at least as high as needed, for all but the most rampant variance and prevalence misspecifications. One limitation is the assumption that the screening test scores have a bivariate normal distribution of the test scores and that the assumptions of the general linear univariate model [22] are met. Secondly, the method may be overly conservative, and result in a Type I error rate lower than nominal. However, for prospective cancer screening trials, being conservative is reasonable. Cancer screening methods may be adopted in large populations, and replicable research is vital for maintaining public trust. Finally, the computing time is somewhat lengthy, because the integration and sums from Equation (10) have high complexity. For any one study design, the amount of time is reasonable. For example, it took less than eight hours to run all programs used in Example 2. In addition, our simulation study demonstrated that the method is not necessary in screening studies with large sample sizes.

## Conclusion

We have shown that an internal pilot approach usually achieves goal power, and, for most studies with sample size greater than 50, requires no Type I error correction. Further, we have provided a flexible and accurate approach to bound Type I error below a goal level for studies with small sample size (*N* < 50). Both investigators and statisticians should use the new methods for the design of cancer screening trials.

## Abbreviations

AUC: area under the receiver operating characteristic curve
MAD: maximum absolute deviation
ELISA: enzyme-linked immunosorbent assay
